# Establishment and identification of immortalized sheep ovarian granulosa cells

**DOI:** 10.1186/s12917-026-05472-1

**Published:** 2026-04-21

**Authors:** Hao Chen, Yue Yuan, Rui Xiao, Xiaona Ding, Zhijie Zhao, Pinshen Li, Bingzhu Zhao, Taojie Zhang, Yingpai Zhaxi, Shengdong Huo

**Affiliations:** College of Life Science and Engineering, Northwest Minzu University, Lanzhou, Gansu 730030 China

**Keywords:** SV40T lentiviral vector, Granulosa cells, Immortalization

## Abstract

**Background:**

Follicular granulosa cells (GCs) are an important cellular resource for studying animal reproductive functions.

**Results:**

This study aimed to investigate SV40T lentiviral vector-mediated gene transfection in sheep ovarian granulosa cells (GCs) and systematically validate their biological characteristics and functions after immortalization. By optimizing infection conditions (MOI = 10), efficient transfection of the GFP-tagged SV40T gene was successfully achieved (approximately 90% efficiency), leading to the establishment of an immortalized sheep granulosa cell line (GCs-SV40T-GFP). This cell line could be stably passaged for over 50 generations while maintaining consistent cellular morphology and stable fluorescence expression. Compared to primary cells, the immortalized cells exhibited significantly accelerated proliferation, with flow cytometry revealing a marked increase in the proportion of cells in S and G1 phases (*P* < 0.05). Functional assays confirmed that these cells continuously secreted estradiol (*P* < 0.05). Further experiments demonstrated that even at the 50th passage, the immortalized cells still highly expressed key granulosa cell functional markers, FSHR and CYP19A1. Karyotype analysis confirmed the maintenance of a normal chromosomal karyotype across different passages (primary, 10th, 25th, and 50th). Additionally, the cells exhibited a dose-dependent functional response to hormonal stimulation, with FSHR protein expression significantly upregulated in response to increasing concentrations of exogenous estradiol (*P* < 0.05). In vivo safety evaluation showed that GCs-SV40T-GFP cells did not induce tumor formation in mice.

**Conclusions:**

In this study, a functionally stable immortalized sheep granulosa cell model was successfully constructed. At the same time, it also provides an important tool for animal reproductive function research and hormone regulation mechanism exploration.

**Graphical abstract:**

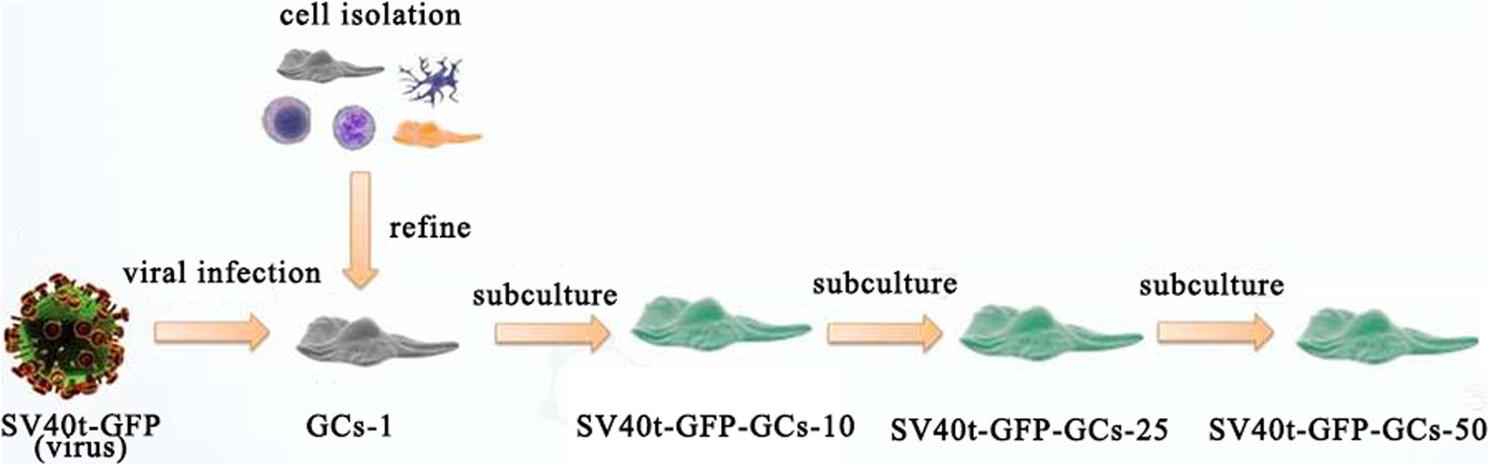

## Introduction

Granulosa cells (GCs) are key functional cells that directly regulate oocyte development and steroid hormone synthesis in mammalian follicles [1]. In the follicular microenvironment, GCs coordinate the physiological processes of follicular growth, atresia or ovulation by secreting hormones such as estradiol and inhibin [2]. However, primary GCs generally have problems such as limited proliferation ability and easy aging in vitro culture, which seriously restricts their application in reproductive biology research [3], such as dynamic monitoring of hormone secretion, gene function analysis or drug screening model construction [4]. Therefore, the establishment of a stable and fully functional immortalized GCs model has become an important direction to break through this technical bottleneck [5].

In comparison to other immortalization strategies, the selection of SV40T as the immortalizing agent in this study is primarily based on its core advantages and mechanistic rationale [6]. First, the simian virus 40 large T antigen (SV40T) efficiently bypasses replicative senescence by inhibiting key tumor suppressor proteins such as p53 and Rb, thereby driving cells into the S-phase and conferring unlimited proliferative potential [7]. This mechanism has been widely validated across various mammalian cell types, including human, murine, and bovine models, demonstrating high reliability and efficiency. Second, lentiviral vector-mediated delivery of SV40T ensures stable genomic integration, sustained transgene expression, and low immunogenicity, which are critical for maintaining long-term cellular function and phenotypic stability [8]. Furthermore, the fusion of SV40T with a GFP reporter allows real-time monitoring of transfection efficiency and expression consistency throughout serial passages—an innovative feature not commonly emphasized in alternative approaches such as telomerase (hTERT) overexpression or oncogene-based methods. While other strategies like hTERT activation may reduce tumorigenic risk, they often require complementary genetic modifications to achieve full immortalization and may not equally enhance proliferation in slow-cycling primary cells like sheep granulosa cells [9]. Thus, the SV40T-lentivirus system offers a balanced combination of high immortalization efficiency, functional preservation, and technical feasibility, making it particularly suitable for establishing a stable and biologically relevant sheep granulosa cell model for reproductive research [10].

Lentiviral vector has become the preferred tool for gene delivery due to its high transfection efficiency, stable genome integration and low immunogenicity [11]. In this study, the lentiviral vector of SV40T and green fluorescent protein (GFP) fusion was constructed, and the multiplicity of infection (MOI = 10) was optimized to achieve efficient transfection of sheep GCs (efficiency of 90%), and an immortalized cell line (GCs-SV40T-GFP) was established [12]. Preliminary experiments confirmed that the transfected cells remained stable in morphology and fluorescence expression after continuous passage to the 50th generation, which preliminarily verified the long-term integration and expression stability of SV40T gene [13].

Although SV40T immortalization technology has significant advantages, its application still faces two major challenges: one is the maintenance of cell function after immortalization [14]. For example, immortalized human mesenchymal stem cells can retain the ability to differentiate, while some hepatocytes have reduced metabolic activity. For GCs, whether the synthesis ability of its core function-steroid hormones (such as estradiol) is impaired directly affects the research value of immortalized cells. The second is the potential tumorigenicity of SV40T [15, 16]. Therefore, this study not only needs to verify the hormone secretion function of GCs-SV40T-GFP, but also needs to evaluate its biosafety through in vivo transplantation experiments [17].

As an important economic animal and biomedical model, the reproductive physiology of sheep is of great significance to the development of animal husbandry breeding and human assisted reproductive technology. However, the short life characteristics of sheep GCs limit the depth of in vitro research [18]. In this study, an immortalized GCs-SV40T-GFP cell line was constructed to provide a stable and functional experimental model for analyzing the potential effects of follicular development mechanism, hormone regulation network and environmental pollutants on reproductive function in sheep [19]. In addition, if the model is proved to be safe and reliable, it can provide technical support for improving animal reproduction efficiency [20].

In summary, this study focused on SV40T lentiviral vector-mediated sheep granulosa cell immortalization technology, and systematically evaluated its proliferation characteristics, functional stability and biosafety, aiming to fill the technical gaps in this field and provide innovative tools for reproductive biology research and clinical application [21].

## Materials and methods

### Treatment of GCs

In this study, 10 ovaries of 2-year-old healthy ewes were collected from the slaughterhouse in Tianzhu County, Gansu Province, and stored in 37° normal saline with penicillin and streptomycin (0.48 g) added to the laboratory. Follicular fluid was extracted from ovarian follicles with a diameter of about 3 ~ 8 mm and injected into the tube. The follicular fluid was sequentially filtered through 100 nm and 70 nm filter membranes. The resulting filtrate was then centrifuged at 1500 r/min using a high-speed centrifuge (TG16-WS benchtop high-speed centrifuge), and the supernatant was discarded. The cell pellet was cultured in DMEM medium supplemented with 10% fetal bovine serum. Cell morphology was observed, and the cells were maintained in a cell incubator at 37 °C in a 5% CO2 atmosphere.

### Cell counting plate method

In this study, sheep granulosa cells were cultured in vitro, and cell proliferation dynamics within 96 h were systematically monitored using the cell counting plate method. The specific procedure was as follows: Synchronized granulosa cells were cultured in DMEM medium supplemented with 10% fetal bovine serum, and samples were collected every 12 h starting from 0 h. The cells were dissociated using trypsin digestion. Viable cells were counted under an inverted microscope using a modified Neubauer counting chamber with trypan blue background staining. Biological and technical replicates were set at each time point. Finally, cell density was calculated, the growth curve was plotted, and the population doubling time was determined.

### Immunofluorescence staining

Primary cultured granulosa cells were seeded in 24-well plates, and the culture was terminated when the cells reached 80% confluence. Then immunofluorescence detection was performed. GCs were fixed with 4% paraformaldehyde for 30 min, and 0.1% Triton X-100 permeabilized the cell membrane for 15 min. It was blocked with 5% BSA (Gibco, Carlsbad, CA) at room temperature for 2 h. Cells were incubated with rabbit monoclonal antibody Vimentin at a concentration of 1:200 (Bioss, Beijing, China) overnight at 4 °C. The cells were incubated with anti-rabbit secondary antibody at a concentration of 1:500 (Bioss, Beijing) and incubated at room temperature for 1 h the next day. Blank control was only incubated with secondary antibody. DAPI was used for nuclear re-staining. Cells were briefly washed three times with PBS between the start, end, and each step of staining. Immunostaining was evaluated by fluorescence microscopy. This experiment was repeated three times.

### Confirmation of GFP-GCs (multiplicity of infection) in sheep granulosa cells

On the first day, SV40T sheep ovarian granulosa cells were inoculated in a 12-well plate containing 1E5 cells. The next day, the GFP virus stock solution ( Cegrogen Shanghai ) was taken out from the − 80 °C refrigerator and melted in an ice bath before infection. The virus GFP stock solution was diluted with 800µL complete medium with MOI = 1,10,50,100, and the original medium of the treatment group was removed. 500µL medium containing lentivirus dilution was added to the cells in the treatment group. The culture medium was changed on the third day, and the culture medium containing lentivirus was changed to 1 m L complete culture medium after 16 h of infection. On the 5th day, the infection efficiency was detected. The fluorescence was observed under an inverted fluorescence microscope, and the effect of lentivirus infection on target cells was calculated by fluorescence intensity.

### SV40T-GFP-GCs lentivirus transfection

At MOI = 10, SV40T-GFP transfection was performed. The resulting immortalized cell line GCs-SV40T-GFP showed no significant differences in estradiol secretion or expression of key markers FSHR and CYP19A1, confirming that continuous antibiotic pressure did not alter the core physiological characteristics of the cell line.

### RNA isolation, reverse transcription and quantitative RT-PCR

Total RNA was extracted using the Trizol method (SOLEIBO, Beijing, China), and the concentration and purity of RNA samples were evaluated using an ultra-micro UV-visible spectrophotometer (NanoReady/FC-1100) spectrophotometer. RNA samples with D260/D280 values between 1.8 and 2.0 were selected for cDNA synthesis using a PrimeTM TMRT kit (Takara, Beijing, China) with 1 µg of high-purity RNA. SYBR PreMix Ex Taq TM II (Takara, Beijing, China) and real-time PCR system (Bio-Rad, Hercules, CA, USA) were used for qRT-PCR. The reaction conditions are as follows: pre-denaturation at 95 °C for 3 min, followed by denaturation at 95 °C for 10 s, and annealing at 60 °C for 30 s, for a total of 40 cycles. Each group consists of three biological replicates. The relative expression was calculated by 2-ΔΔCt. According to the gene sequence of sheep in GenBank database, primers were designed by Primer Premier 5.0 software for real-time fluorescence quantitative PCR. The primers were synthesized by Beijing Huada Gene Information Company (Table 1).

**Table 1:** GCs-SV40T Real-time PCR primer sequences

Gene name	Sequences (5’→3’)	Length(bp)	Accession number
CYP19A1	F-ATGCTGGTGCTGAGTATGTGGT	192	NM_001123000.1
	R-GCTGACAATCTTGAGGGTGTTG		
SV40T	F-TGGAAACCAAGTGCGACGAC	671	NC_001669.1
	R-CGGCGAAGATAGCGGCATTA		
ACTB	F-ATTGCTGACAGGATGCAGAAGG	225	NM_001101.5
	R-GCTGGAAGGTGGACAGTGAGG		
FSGR	F-TCATGGGACTGAGCTTTGAAAGT	246	NM_013523.3
	R-TGGCCCTCAACTTCTTCAGATTT		
ACTIN	F-ATATTGCTGCGCTCGTGGTT	224	NM_001009784.3
	R-GTTGGTGACAATGCCGTGCT		

### Flow cytometry

GCs-1, SV40T-GFP-GCs-1, SV40T-GFP-GCs-10, SV40T-GFP-GCs-25, SV40T-GFP-GCs-50 were added to 200µL cell suspension and added to 6-well plates. Add 10% fetal bovine serum complete medium, pre-incubated at 37 °C, 5% CO2 incubator for 48 h. Rise to about 90%, trypsin digestion and centrifugation. The stained cell suspension was transferred to a flow tube, and the data were detected and analyzed by flow cytometry.

### ELISA detection

In order to reduce the influence of fetal bovine serum, phenol red-free medium was used to avoid estrogen interference, and activated carbon/dextran-treated double-antibody FBS was replaced. Before the formal experiment, 24-hour serum starvation was performed and replaced with 0.5-1% stripped FBS medium. 28.6ng/m LA4 (androstenedione) was added. The primary, 10th, 25th and 50th generations were recorded respectively. When they grew to 100% of the cells, the complete medium was collected in a 1.5mL centrifuge tube. After centrifugation at 3000 rpm for 15 min, the supernatant was taken. Concentrations were determined using kits (Shanghai, China, Mlbio).

### Western blot assay

The treated cells were collected and lysate and PMSF (100:1, Servicebio, China) were added to the cell precipitate. Lysis was performed on ice for 30 min followed by centrifugation at 10,000×g at 4℃for 15 min. Protein concentration was determined using the BSA kit (Servicebio, China). The protein loading volume was 50 mg. Separation was performed by electrophoresis using 10% SDS-PAGE followed by electrotransfer to polyvinylidene difluoride membranes (MCE, New Jersey, USA). The membranes were closed in 5% skim milk in TBS/Tween for 1 h at room temperature and then mixed with FHSR(Servicebio, China) 1:2000 and β-actin (Servicebio, China) 1:3000 with skim milk-PBST (Servicebio, China) and co-incubated. The membranes were then incubated with HRP conjugated antirabbit or anti-mouse secondary antibody 1:1000 (Servicebio, China) with 5% skim milk-PBST and detected by the Western Lighting ECL detection system. Signals were quantified using Image J 2020.

### Tumorigenicity experiment in mice

Nine 5-week-old male BALB/c nude mice were purchased from Lanzhou Veterinary Research Institute and randomly divided into 3 groups with 3 mice in each group. After 1 week of adaptive culture, primary granulosa cells, 50th generation immortalized granulosa cells and HeLa cells were inoculated respectively. The cells were placed in two T25 culture flasks and cultured in DMEM medium containing 20% fetal bovine serum. The incubator was set at 37 °C, 5% carbon dioxide. Until the cell growth is greater than 98%. After trypsin digestion, each generation of cells was adjusted to 1 × 7 Ωcells / mL, and 10 µL was injected subcutaneously into the left back of mice. After 4 weeks of continuous observation, the tumor size was monitored with a ruler. Euthanasia via cervical dislocation.

### Karyotype analysis

Primary, 10th, 25th, and 50th passage cells were selected for karyotype analysis. Cells in the logarithmic growth phase were cultured in 6-well plates and treated with 1 µg/mL colchicine for 4–6 h to arrest them at metaphase by inhibiting spindle formation. After trypsin digestion, the cells were collected and centrifuged at 1000 rpm for 8 min. The pellet was resuspended in pre-warmed 0.075 M KCl hypotonic solution and incubated at 37 °C for 20 min. After centrifugation, the supernatant was discarded, and the cells were fixed with freshly prepared methanol: glacial acetic acid (3:1) fixative at room temperature for 30 min. The fixation step was repeated twice, followed by resuspension in fresh fixative. The cell suspension was dropped onto pre-chilled glass slides and air-dried. Slides were stained with Giemsa solution for 10–15 min, rinsed with distilled water, and air-dried. Chromosome morphology was observed under an optical microscope (100× oil immersion objective), and at least 50 complete metaphase spreads per sample were analyzed to determine chromosome numbers statistically.

### Statistical analysis

All data represent at least three independent experiments (with comparable results). ANOVA was used to evaluate the statistical significance of the data. The difference of *P* < 0.05 was considered statistically significant. One-way analysis of variance was used to analyze the data using GraphPad Prism 8 software, and paired comparisons were performed using LSD and Tukey tests. The results are expressed as mean ± standard deviation.

## Results

### Figure 1 Establishment of primary sheep granulosa cell cultures

**Fig. 1:**
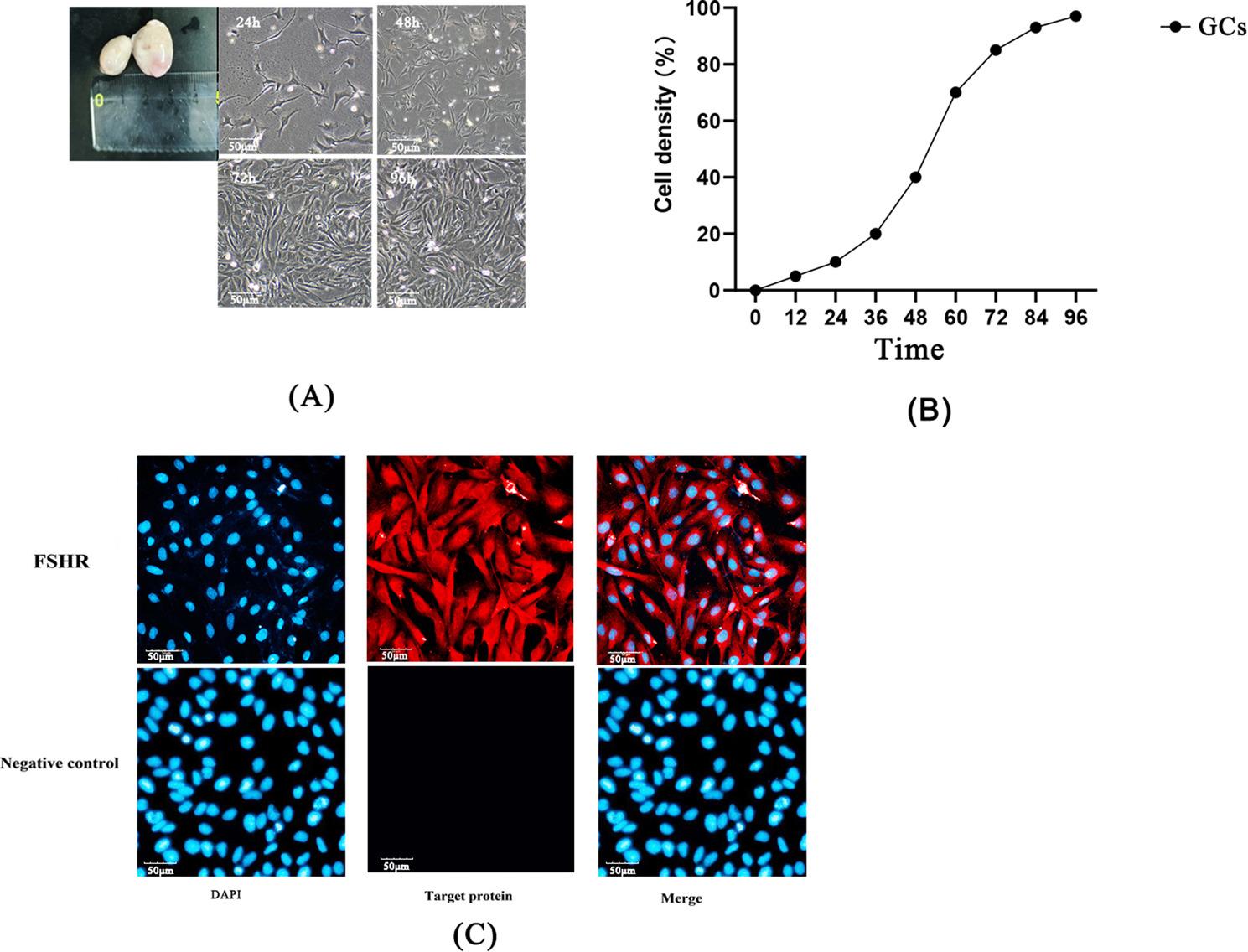
Culture and identification of the first generation of GCs cells: (**A**) Cell growth and identification. **B** Cell growth time and density (**C**) Immunofluorescence detection of fshr in GCs cells

Granulosa cells (GCs) were isolated from the ovarian follicles of healthy ewes to establish an in vitro model (Fig. 1A). The primary cells exhibited an epithelial-like morphology and formed confluent monolayers. A 96-hour growth curve revealed a typical sigmoidal proliferation pattern, confirming their robust proliferative capacity (Fig. 1B). Immunofluorescence staining was performed for follicle-stimulating hormone receptor (FSHR), which verified their identity as granulosa cells (Fig. 1C). This characterization provided a well-defined cellular foundation for subsequent experiments.

### Figure 2 Generation of immortalized granulosa cell lines

**Fig. 2:**
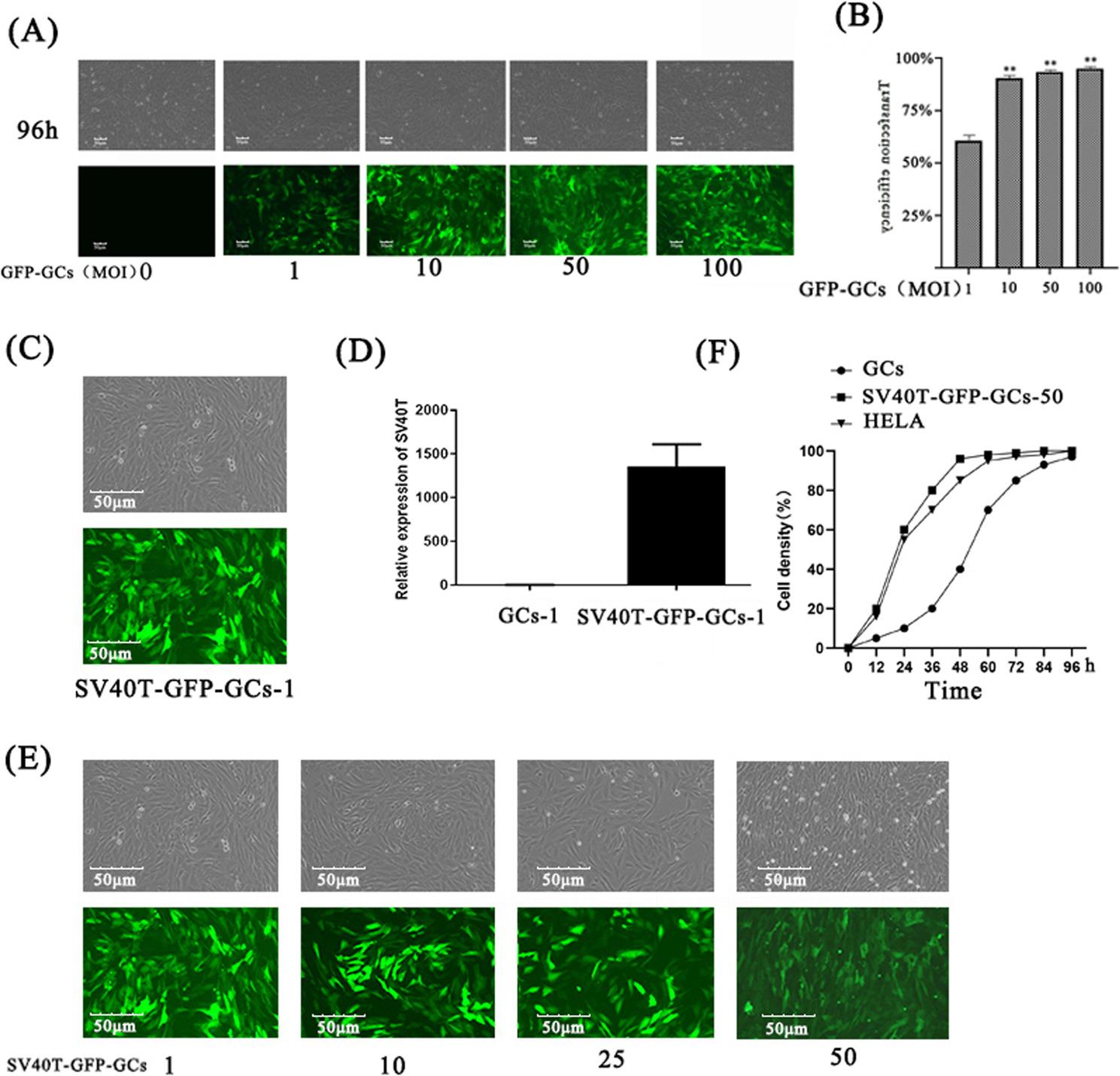
Establishment of immortalized cells: (**A**-**B**) Different MOI concentrations (0, 1, 10, 50, 100) were tested. At an MOI of 10, the transfection efficiency reached 90%, which was suitable for subsequent experiments (*P* < 0.05). **C** Primary granulosa cells (GCs) cultured for 96 hours were transfected to obtain the first passage of SV40T-GFP-GCs-1 cells, which exhibited obvious fluorescence expression. **D** qPCR analysis showed that SV40T-GFP gene expression was significantly higher in SV40T-GFP-GCs-1 cells compared with GCs-1 cells. **E** During subsequent passages, cell morphology remained unchanged, and SV40T-GFP gene continued to show high fluorescence expression. **F** The cell growth time after transfection was significantly shortened (*P* < 0.05)

At an MOI of 10, approximately 90% of the cells were GFP-positive (Fig. 2A, B). Higher MOI values (50 and 100) did not further improve transduction efficiency. Therefore, an MOI of 10 was selected for subsequent transductions to achieve high efficiency. Primary GCs were transduced with SV40T-GFP lentivirus at an MOI of 10. The cells exhibited strong GFP fluorescence (Fig. 2C), and qPCR confirmed significantly upregulated expression of SV40T (Fig. 2D). Growth curve analysis showed that, compared with primary GCs, the immortalized cells entered the exponential growth phase earlier and exhibited a significantly reduced doubling time (Fig. 2F). The cell line maintained stable morphology across different passages SV40T-GFP-GCs-1, SV40T-GFP-GCs-10, SV40T-GFP-GCs-25, and SV40T-GFP-GCs-50;(immortalized passages 1, 10, 25, and 50), with consistent GFP expression observed in all passages (Fig. 2E), indicating that the immortalized cells possess stable passage capability. Bars with different letters are statistically different (*P* < 0.05).

### Figure 3 Generation of immortalized granulosa cell lines

**Fig. 3:**
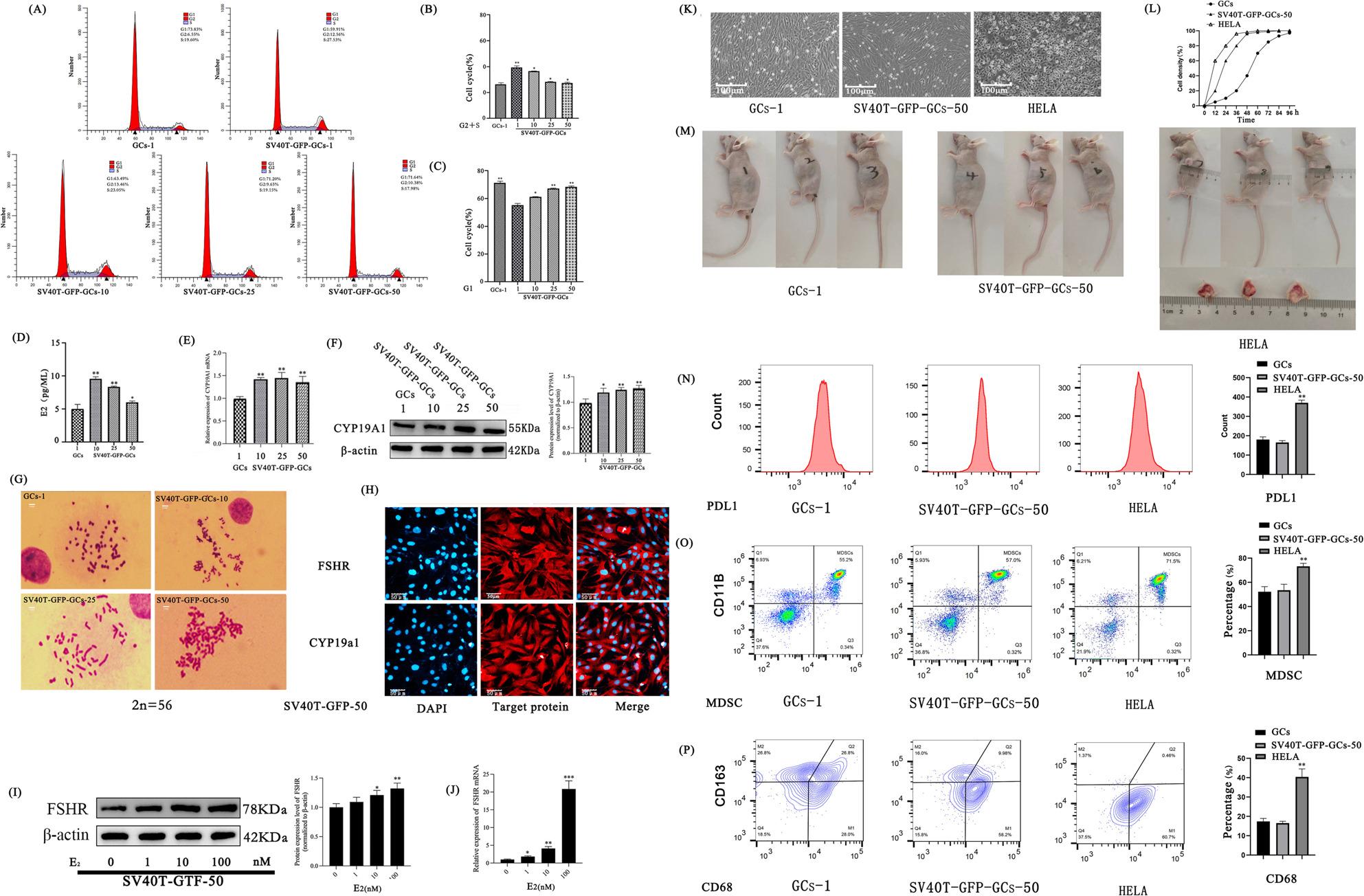
Characterization of the immortalized granulosa cell line: (**A**–**C**) Compared with GCs-1, the proportion of SV40T-GFP-GCs in the S+G1 phase was significantly increased in the 1st and 10th passages after transfection, indicating markedly enhanced proliferative activity (*P* < 0.05). **D** Compared with GCs-1, estradiol levels were significantly elevated in the SV40T-GFP-GCs-10 and SV40T-GFP-GCs-25 groups. **E**, **F** CYP19A1 protein expression was detected by Western blot and quantitative PCR (qPCR). **G** Karyotype analysis demonstrated that cells maintained normal chromosomal morphology across all passages (primary, 10th, 25th, and 50th generations). **H** The 50th passage immortalized cells sustained high expression of the key granulosa cell functional markers FSHR and CYP19A1. **I**–**J** Western blot and qPCR analyses confirmed a dose‑dependent upregulation of FSHR protein expression in response to increasing concentrations of exogenous estradiol, with statistically significant differences between groups labeled with different letters (*P* < 0.05). **K** Cell morphology. **L** Growth density. **M** Mice inoculated with HeLa cells developed obvious tumors, whereas no tumors were observed in the GCs-1 or SV40T-GFP-GCs-50 groups. **N**–**P** MDSC, PD‑L1, and CD68 cells were significantly increased (*P* < 0.05)

Flow cytometric analysis revealed that, compared with primary granulosa cells (GCs-1), the proportion of SV40T-GFP-GCs in the S phase was significantly increased in the 10th passage (Fig. 3A-C). This altered cell cycle pattern, characterized by accelerated progression into the DNA synthesis phase, was consistently maintained in later passages (25th and 50th), indicating that SV40T immortalization promotes increased proliferative activity. ELISA analysis showed that estradiol secretion was significantly higher in the 10th and 25th passages of SV40T-GFP-GCs compared with primary GCs (Fig. 3D). Western blot and qPCR confirmed significantly elevated expression of CYP19A1 (Fig. 3E, F), suggesting preserved functionality. Karyotype analysis revealed that cells at all passages maintained a normal diploid karyotype (2n = 54) (Fig. 3G). Strong expression of FSHR was observed in passage 50 cells (Fig. 3H). Stimulation of passage 50 cells with estradiol induced a dose-dependent upregulation of FSHR expression (Fig. 3I, J), demonstrating intact hormone responsiveness. Primary GCs, passage 50 SV40T-GFP-GCs, and HeLa cells were inoculated into immunodeficient mice (Fig. 3K-L). After 4 weeks, obvious tumors formed exclusively in the HeLa group (Fig. 3M), whereas no tumors were observed in either the primary GC group or the passage 50 SV40T-GFP-GCs group. Flow cytometric analysis revealed that only the HeLa group exhibited significant increases in MDSC, PD-L1+,and CD68 + cells in mouse blood (Fig. 3N-P), indicating that SV40T-GFP-GCs lack tumorigenic potential under these conditions. Bars with different letters are statistically different (*P* < 0.05).

## Discussion

In this study, an immortalized sheep granulosa cell line (GCs-SV40T-GFP) was successfully constructed by SV40T lentiviral vector, and its proliferation characteristics, functional stability and biosafety were systematically evaluated [22]. The experimental results are discussed from four aspects: technical advantages, function maintenance mechanism, security disputes and future application prospects [23].

In this study, the transfection efficiency of SV40T gene mediated by lentiviral vector was as high as 90% (MOI = 10), and the transfected cells still maintained stable morphology and fluorescence expression after continuous passage to the 50th generation [24]. This result highlights the significant advantages of lentiviral vectors in gene delivery: their genomic integration ensures long-term expression of foreign genes, while low immunogenicity reduces interference with cell activity. Compared with traditional plasmid transfection or retrovirus, the ability of lentivirus to infect non-dividing cells further improves its application potential in primary cell immortalization. In addition, the introduction of GFP tags provides an intuitive tool for real-time monitoring of transfection efficiency and gene stability during cell passage. This design is innovative in similar studies [25].

It is worth noting that SV40T significantly enhances the proliferation of GCs by targeting p53 and Rb proteins to relieve cell cycle arrest [26]. Cell cycle analysis showed that the proportion of S phase cells in GCs-SV40T-GFP increased, which was consistent with the results of human immortalized mesenchymal stem cells. However, the response of sheep GCs to SV40T may be species-specific [27]. For example, the increase in cell proliferation rate in this study was higher than that in some bovine or mouse cell models, which may be related to the low original proliferation potential of sheep GCs. This finding suggests that the effect of SV40T immortalization is not only dependent on gene delivery efficiency, but also closely related to the biological characteristics of target cells [28].

Although cellular immortalization is often accompanied by functional abnormalities, this study found that GCs-SV40T-GFP cells continued to secrete estradiol at levels comparable to those of primary cells, with no significant difference in secretion levels [29]. However, some immortalized hepatocytes have been observed to exhibit reduced metabolic enzyme activity after passaging, which may be associated with the accumulation of epigenetic modifications or mitochondrial dysfunction. Therefore, it is necessary to conduct transcriptomic or metabolomic analyses in the future to gain an in-depth understanding of the overall impact of SV40T on signaling pathways in sheep granulosa cells [30].

In addition, the secretion dynamics of other hormones (such as progesterone or inhibin) were not detected in this study. Sheep GCs have functional heterogeneity at different developmental stages of follicles, and whether immortalization causes cells to ' lock ' into specific functional states remains to be verified. If GCs-SV40T-GFP can still respond to gonadotropin (such as FSH) stimulation and regulate hormone synthesis, the model can be used to simulate the dynamic process of follicular development and has higher research value [31].

The risk of tumorigenicity of SV40T is the core issue that limits its clinical application. In this study, we confirmed that GCs-SV40T-GFP did not induce tumor formation by in vivo transplantation experiments in mice. This result contradicts some studies: for example, SV40T immortalized human fibroblasts can form sarcomas in immunodeficient mice [32]. The difference may be due to the following reasons: (1) Species specificity: the tumorigenic transformation threshold of sheep GCs may be higher than that of human cells; (2) Gene expression regulation: The promoter activity or copy number control of SV40T in lentiviral vector may reduce its carcinogenic potential. (3) Cell type difference: GCs themselves are terminally differentiated cells, and their malignant transformation requires additional driver mutations. Nevertheless, long-term safety still needs to be carefully evaluated. It is recommended that follow-up studies extend the observation period and use more sensitive detection methods (such as circulating tumor DNA analysis) to exclude the possibility of small lesions [33].

The GCs-SV40T-GFP cell line established in this study provides a valuable tool for sheep reproductive biology research, addressing the current gap in immortalized granulosa cell models for this species. When compared to existing immortalized granulosa cell models in cattle, pigs, and dogs, the immortalization efficiency and functional maintenance achieved in sheep in this study reach comparable levels. For instance, while SV40T-mediated immortalization in bovine granulosa cells similarly preserves hormonal secretion capacity, the proliferation rate observed in bovine models is lower than that in our sheep model, indicating species-specific differences in responses to immortalizing genes. Additionally, whereas immortalized porcine granulosa cells often exhibit functional decline during serial passaging, the cell line developed in this study maintains stable estradiol secretion during long-term culture, demonstrating superior functional sustainability [13].

In terms of applications, this cell line can be utilized to: (1) elucidate the regulatory mechanisms of key genes (e.g., FOXL2, AMH) in sheep follicular development and perform cross-species comparisons with cattle, dogs, and other species; (2) screen natural compounds or pharmaceutical agents that modulate steroid synthesis, thereby providing species-specific data for reproductive endocrine studies; (3) assess the toxic effects of environmental pollutants on ovarian function in sheep, helping to address the shortage of reproductive toxicology models for large domestic animals. In livestock production, this model can also be applied to optimize oocyte maturation conditions in sheep in vitro fertilization systems or to develop high-throughput screening platforms for efficient ovulation-inducing drugs tailored to sheep. Compared to model animals such as dogs, sheep, as monotocous large livestock, exhibit follicular development patterns and hormonal regulatory mechanisms more closely aligned with those of humans, thereby highlighting the potential value of this cell line in translational medicine. In the future, by integrating gene-editing technologies such as CRISPR-Cas9, specific gene mutation models could be further engineered in the GCs-SV40T-GFP cell line to investigate the molecular mechanisms underlying reproductive disorders, thereby advancing research in reproductive physiology of large domestic animals [6].

## Conclusions

This study confirmed that SV40T lentiviral vector can efficiently realize the immortalization of sheep granulosa cells, and the immortalized cells meet the basic research needs in terms of proliferation ability, functional characteristics and biosafety. However, the following issues still need to be further explored: (1) The effect of immortalization on the epigenetic characteristics and stress response ability of GCs; (2) The functional recovery efficiency of cell lines after long-term cryopreservation and resuscitation; (3) Synergistic effect of SV40T with other immortalized genes (such as hTERT). Future work can combine multi-omics technology with in vitro and in vivo functional verification to comprehensively evaluate the reliability of the cell line and expand its practical application in reproductive medicine and animal husbandry. In conclusion, the establishment of GCs-SV40T-GFP cell line not only fills the technical gap in the in vitro research model of sheep germ cells, but also lays a solid foundation for further exploring the regulation mechanism of follicular development and developing new reproductive intervention strategies.

## Data Availability

The data that support the findings of this study are available from the corresponding author upon reasonable request.

## Abbreviations

AMH: Anti-Müllerian Hormone
SDS-PAGE: Sodium Dodecyl Sulfate–Polyacrylamide Gel Electrophoresis
DAPI: 4′,6-Diamidino-2-Phenylindole
DNA: Deoxyribonucleic Acid
ELISA: Enzyme-Linked Immunosorbent Assay
FOXL2: Forkhead Box L2
GCs: Granulosa Cells
hTERT: Human Telomerase Reverse Transcriptase
PBS: Phosphate Buffered Saline
qRT-PCR: Quantitative Reverse Transcription Polymerase Chain Reaction
StAR: Steroidogenic Acute Regulatory Protein
BSA: Bovine Serum Albumin
CYP19A1: Cytochrome P450 Family 19 Subfamily A Member 1
DMEM: Dulbecco’s Modified Eagle Medium
ECL: Enhanced Chemiluminescence
FBS: Fetal Bovine Serum
FSH: Follicle-Stimulating Hormone
GFP: Green Fluorescent Protein
MOI: Multiplicity of Infection
cDNA: Complementary DNA
SV40T: Simian Virus 40 Large T Antigen
